# Development and applications of graduate outcome-based curriculum for basic medical education

**DOI:** 10.3389/fmed.2024.1400811

**Published:** 2024-08-16

**Authors:** Guang Chen, Hongmei Wang, Lingling Zhou, Jingjin Yang, Linglong Xu, Yong Liang

**Affiliations:** ^1^Undergraduate Teaching Office, School of Medicine, Taizhou University, Taizhou, Zhejiang, China; ^2^Undergraduate Teaching Office, Taizhou Central Hospital (Taizhou University Affiliated Hospital), Taizhou, Zhejiang, China

**Keywords:** graduate outcome-based curriculum, outcome-based education, Chinese medical education reform, curriculum, medical education

## Abstract

**Introduction:**

Outcome-based education (OBE) leads to revolutionary change in medical education, but each medical school is acknowledged to develop suited framework based on specific positioning, educational philosophy and expected outcomes.

**Methods:**

Based on the graduate outcomes of basic medical education in China released by Working Committee for the Accreditation of Medical Education (WCAME) which officially recognized by World Federation for Medical Education (WFME), Taizhou University re-documented the overall organization of the curriculum and classified the courses as “Crucial, Supporting and Associated (CSA)” categories to connect the graduate outcomes and course objectives.

**Results:**

We redefined the standard for graduates in Taizhou University Medical School including 34 items in four domains and redesigned the curriculum with 76 courses classified as CSA. Empirical data reveals a substantial improvement in students’ performance on Standardized Competence Test for Clinical Medicine Undergraduates in China (SCTCMU) by implementing the CSA system between 2022 and 2023. Notably, in 2023, Taizhou University’s students achieved pass rates more than 20 percentage points higher than the national average, demonstrating a profound and unprecedented impact.

**Conclusion:**

The CSA classification system provides a notably clear and structured framework for categorizing courses based on their direct or indirect relevance to educational objectives, which provides clarity to educators and empowers students with a more purposeful approach to their studies.

## Highlights

- Medical education must define the curriculum models.
- Medical curriculum should connect graduate outcomes and course objectives.
- The CAS system deepens the impact of the curriculum.

## 1 Introduction

Medical education standards and guidelines are essential for maintaining global consistency in medical training (1). Organizations such as the World Health Organization (WHO), the International Federation of Medical Students’ Associations (IFMSA), and the Association for Medical Education in Europe (AMEE) provide valuable direction for medical student education. However, disparities in medical education persist across countries due to variations in culture, healthcare systems, and regulatory frameworks (2). The “Global Standards for Quality Improvement,” established by the World Federation for Medical Education (WFME), serve as a comprehensive guide for medical education worldwide, covering critical domains such as curriculum, assessment, faculty development, and quality enhancement (3).

In China, as in many nations, there is a compelling need to strike a balance between adhering to global standards and addressing the unique requirements of the national healthcare and educational landscape (4), though Working Committee for the Accreditation of Medical Education (WCAME) was officially recognized by the World Federation for Medical Education (WFME), marking that China’s medical education standards and accreditation system have achieved international substantive equivalence, and the quality of medical education accreditation has been internationally recognized. The COVID-19 pandemic, which underscored the unprecedented challenges facing the healthcare sector, has laid bare the shortcomings of the existing medical education curriculum in China. The rapidly changing healthcare landscape, the need for telemedicine, infectious disease management, and an increased focus on public health have become critical facets of medical practice. This new reality highlights the urgency of reevaluating and updating the medical curriculum to better prepare students for the multifaceted demands of modern healthcare.

Despite significant advancements in China’s medical education system, it faces several challenges. The rapid proliferation of medical schools has raised concerns about the consistency and quality of education (5). Variability in the quality of medical education across different Chinese institutions is a significant concern (6). The central challenge is to maintain curriculum relevance and ensure alignment with the evolving requirements of China’s healthcare system and society. Aligning the national curriculum with global standards while addressing local healthcare needs and practices is complex. This integration requires continuous updates and reforms to keep pace with global advancements. Training and retaining qualified faculty who are well-versed in both traditional practices and modern educational techniques is essential for maintaining high educational standards. Achieving this complex endeavor necessitates continuous attention.

Our novel introduction of the “Crucial, Supporting and Associated” (CSA) classification system stands as a testament to China’s commitment to addressing the challenges of its medical education landscape (7) which ensures that students are well-prepared to meet the multifaceted demands of modern healthcare practice. CSA’s adaptability to China’s national conditions, alignment with Outcome-Based Education (OBE), and emphasis on well-rounded development underscore its remarkable role in enhancing medical education. This forward-thinking system is poised to foster a new generation of healthcare professionals who are not only skilled and adaptable but also deeply attuned to the evolving needs of China’s healthcare system.

## 2 Method

### 2.1 Optimizing the logic of course setting: a reform for enhanced medical education in China

In response to the complex challenges within China’s medical education system, a significant transformation has been initiated to redefine the logic of course setting. This transformation seeks to not only streamline the curriculum structure but also introduce a novel and innovative classification. We developed a new classification system, featuring “Crucial (C), Supporting (S), and Associated (A)” categories, is poised to revolutionize the medical education landscape by offering a more purposeful and structured approach.

The fundamental basis for this classification revolves around the pivotal relationship between courses and their direct support of graduate outcomes. The introduction of the “Crucial, Supporting, Associated” classification system represents a fundamental shift in how courses are integrated into the medical curriculum. It aims to bring greater clarity to the roles and contributions of each course within the broader educational objectives. This strategic realignment ensures that every course serves a specific purpose, fostering a more coherent and goal-oriented educational experience, as shown in the Supplementary Table S1.

#### 2.1.1 Category C: crucial course, the educational backbone

The inception of the Category C course was born out of a profound understanding of the evolving landscape of medical education. It was designed with a clear intent to create a learning experience that aligns perfectly with OBE principles. The fundamental purpose was to equip future healthcare professionals with the knowledge, skills, and competencies needed to excel in a dynamic healthcare environment.

At its core, the Category C course embodies the essence of comprehensive medical education. It emphasizes not just the acquisition of knowledge but the mastery of essential competencies. The concept revolves around nurturing a deep understanding of subjects critical to medical practice, such as pharmacology, diagnosis, and therapeutic capabilities. These subjects are not merely part of the curriculum; they are the foundation upon which a successful medical career is built.

The Category C course is synonymous with educational outcomes of the highest order. It is the conduit through which students gain in-depth expertise in core medical disciplines, leading to a profound grasp of patient care and the advancement of medical science. Graduates who successfully complete Category C courses emerge not just as qualified medical professionals but as leaders capable of making a substantial impact on healthcare delivery and the evolution of the medical field.

In an OBE framework, the Category C course is the linchpin that ensures that students acquire the targeted knowledge and skills defined by learning outcomes. It stands alone as a crucible for instilling the fundamental proficiencies needed in medical practice. This course, individually, encapsulates the critical elements of Outcome-Based Education, guiding students through a journey of mastering core competencies, which ultimately prepares them to meet the demanding standards of modern healthcare.

#### 2.1.2 Category S: supporting course, the prerequisite bridge

The conception of the Category S course was grounded in the recognition that a dynamic and modern medical education requires a balanced approach. Its design was driven by the intention to bridge the gap between foundational knowledge and specialized competencies, creating a versatile and adaptive learning experience that harmonizes with the principles of Outcome-Based Education (OBE).

The Category S course is not just a component of the curriculum; it is the linchpin that connects foundational understanding with the pursuit of specialized expertise. Its concept revolves around establishing a strong knowledge base in fundamental subjects while laying the groundwork for more advanced and specialized learning. This course addresses the vital need for students to have a strong foundational understanding of subjects that underpin medical practice.

The Category S course is pivotal in achieving educational outcomes that align with the OBE philosophy. It equips students with a robust foundation in fundamental medical disciplines, ensuring that they comprehend the core principles that govern healthcare. Graduates who successfully complete Category S courses emerge with a solid grounding in the essentials of medical practice and a readiness to advance to more specialized learning. In the context of OBE, the Category S course holds a unique position.

#### 2.1.3 Category A: associated course, enriching the educational experience

The Category A course was conceived with the deliberate intention of providing students with a comprehensive understanding of the broader healthcare landscape. Its creation was guided by the recognition that a modern medical education should extend beyond the confines of specialized subjects. Category A courses represent a paradigm shift in medical education. They transcend the boundaries of specialization and delve into interdisciplinary subjects that encompass the entire spectrum of healthcare. The concept revolves around nurturing a well-rounded understanding of healthcare delivery, ethics, and the broader societal context in which medicine operates. These subjects are not peripheral; they are integral to producing well-informed and socially responsible healthcare professionals.

Category A courses are instrumental in achieving educational outcomes that mirror the OBE philosophy. They provide students with a holistic perspective on healthcare, cultivating a sense of responsibility, ethics, and a deep understanding of healthcare systems. Graduates who successfully complete Category A courses emerge not only as specialists but as professionals who appreciate the multifaceted aspects of healthcare, positioning them to be more socially conscious and adaptable in their careers. This course stands as the educational compass that aligns with global standards and prepares students to navigate the complexities of modern healthcare with a broader, more inclusive perspective.

## 3 Results

### 3.1 Initial achievements of the CSA classification system in Chinese medical education

**Figure 1:**
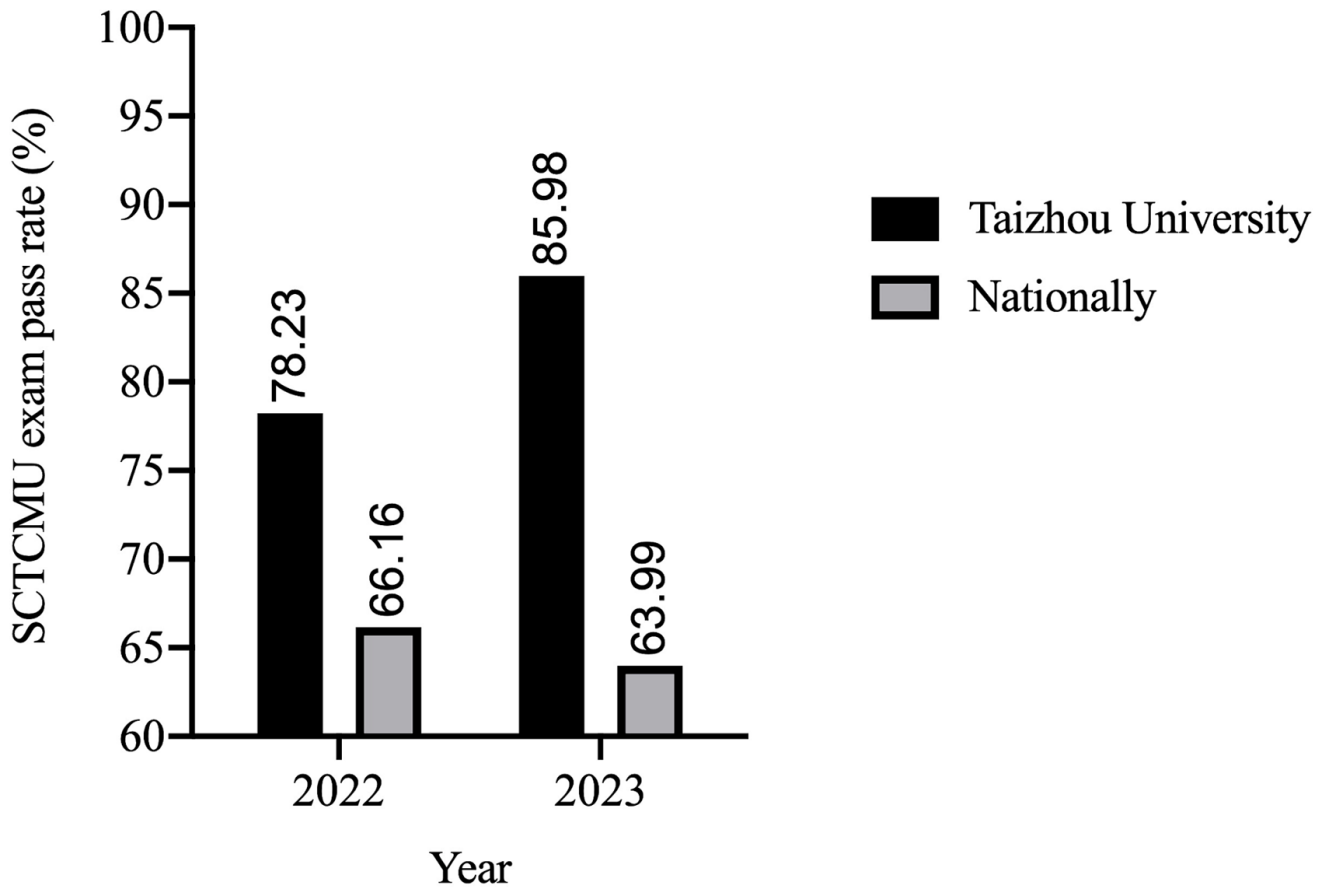
Annual Standardized Competence Test for Clinical Medicine Undergraduates (SCTCMU) total pass rate at Taizhou University and nationally in 2012 and 2023. It presents the yearly SCTCMU total pass rate data for Taizhou University between 2022 and 2023. The pass rate is calculated as the percentage of students who successfully passed their annual examinations.

The implementation of the CSA system in Taizhou University’s medical education curriculum has demonstrated a significant positive impact on the performance of undergraduate students in the Standardized Competence Test for Clinical Medicine Undergraduates (SCTCMU) which is a summative assessment administrated prior to students’ clerkship in China (8). In the years following the adoption of the CSA system, there has been a notable upward trend in the total exam pass rates. In 2022, prior to CSA implementation, the pass rate of students recruited in 2018 from Taizhou University was 78.23%. However, with continued integration and refinement of the CSA system, a distinct improvement was observed in the next year. In 2023, the pass rate students recruited in 2019 when the CAS system implementing was 85.98% (Figure 1; Supplementary material). These outcomes underscore the CSA system’s effectiveness in enhancing learning outcomes and preparing students for success in the medical profession, reflecting the positive impact of this innovative educational approach.

In a comparative analysis of Taizhou University’s medical students’ performance in the SCTCMU against national averages, it is evident that the institution’s implementation of the CSA system has led to a remarkable and unprecedented increase in SCTCMU written exam pass rates. In 2022, the school’s pass rate exceeded the national average, with a pass rate of 78.23% compared to the national rate of 66.16%. However, the most remarkable transformation occurred in subsequent years following the adoption of the CSA system. In 2023, the school’s pass rate was 85.98%, outperforming the national average of 63.99%. Such a substantial and consistent improvement had been observed following the implementation of the CSA system, emphasizing its unprecedented impact on student success.

The implementation of the CSA classification system in Taizhou University’s medical education curriculum has brought about transformative outcomes, significantly enhancing student performance in the SCTCMU. These results underscore a remarkable shift in the institution’s ability to prepare medical students for the rigorous examination. The data reveals that, following the introduction of the CSA system, the total pass rates in 2022 and 2023 surged dramatically, consistently surpassing the national averages by more than 20 percentage points. Such substantial improvements had not been previously witnessed, highlighting the system’s exceptional contribution to academic success and positioning Taizhou University as a trailblazer in medical education. These outcomes signal the CSA system’s ability to not only align with Outcome-Based Education principles but also drive tangible and unprecedented improvements in student achievements, setting a new standard in medical education excellence.

## 4 Discussion

In the present study, we embark on an exploration of the CSA classification system and its seamless integration with the OBE framework. We will delve deeper into how this groundbreaking system not only classifies courses but also shapes an educational environment that prepares students to meet the evolving challenges of the healthcare field. The CSA-OBE partnership is poised to usher in a new era of medical education in China-one that aligns with global standards and adapts to the unique needs of the nation. As we navigate this transformative journey, we embrace the idea that throwing a brick to attract new ideas is the foundation for innovation and educational excellence. Moreover, CSA system might be adapted or applied in different educational or cultural contexts outside of China, upon identifying the graduate outcomes of basic medical education.

The Previous High, Medium, and Low Curriculum Classification offered a comprehensive perspective of the level of each course’s relationship to educational objectives. This granularity allowed educators to have a more nuanced understanding of their courses. Furthermore, it provided a degree of flexibility, enabling educators to adapt and evolve their teaching based on changing educational needs. Nevertheless, the complex nature of this classification system could render it less intuitive and more challenging for educators to navigate and prioritize. The lack of a clear learning path for students regarding how courses relate to their overall education was a notable drawback. Additionally, educators might have found it challenging to allocate resources efficiently when the relationships between courses were less explicitly defined.

The implementation of the CSA classification system represents a paradigm shift with several commendable advantages. It provides a notably clear and structured framework for categorizing courses based on their direct or indirect relevance to educational objectives. This classification system ensures that Category C courses, often regarded as the backbone of medical education, directly address core teaching purposes. Students are systematically guided through a structured learning path, allowing for a more intuitive understanding of the sequence and dependencies between courses. This structure not only provides clarity to educators but also empowers students with a more purposeful approach to their studies.

Furthermore, the CSA system contributes to more efficient resource allocation within educational institutions, enabling educators to focus their teaching efforts where they are most needed. By emphasizing the foundational prerequisites provided by Category S courses, students are better prepared to engage in the more specialized and directly relevant courses within Category C, which is of particular significance for clinical practice. However, it is important to acknowledge a potential pitfall of this classification system. There is a risk of overemphasizing the directly relevant courses (Category C) to the detriment of the broader interdisciplinary aspects of medicine offered by Category A courses. A well-rounded medical education should encompass both specialized and interdisciplinary learning.

In addition, the traditional classification, characterized by simplicity and familiarity, often lacks precision in defining learning outcomes and aligning with the principles of OBE. While it offers ease in curriculum allocation, it may lead to underdevelopment of specialized knowledge and limited adaptability to evolving healthcare demands. In contrast, the CSA classification system in Chinese medical education, when critically evaluated, shines as a flexible and outcome-driven framework that aligns harmoniously with the principles of OBE. It distinguishes itself by adapting to China’s national healthcare conditions, where the need for specialized expertise and a comprehensive understanding of healthcare coexists. This system promotes the cultivation of well-rounded medical professionals who possess the essential knowledge and skills for their field (Category C), as well as the broader perspective needed to navigate the multifaceted challenges of modern healthcare (Categories S and A).

The CSA classification system presents distinct advantages by harmonizing with OBE principles, adapting to the nation’s specific healthcare context, and nurturing well-rounded healthcare professionals. This flexible framework, however, may introduce complexity in implementation, potentially leading to over-categorization and resistance to change among faculty and staff. Effective quality assurance mechanisms are imperative to ensure consistent educational quality across different course categories.

The traditional classification system in Chinese medical education, with its extensive categories of “High, Medium, and Low,” provided simplicity and familiarity in curriculum structuring. It allowed for a straightforward allocation of courses but lacked the granularity and alignment with OBE that the CSA system offers. While the old system offered ease of understanding, it often fell short in defining clear learning outcomes and competencies, hindering students’ preparedness for the dynamic healthcare landscape. CSA, on the other hand, offers the benefits of adaptability, well-rounded education, and OBE alignment, albeit with initial complexities in implementation and potential resistance to change from traditional educational norms.

In the realm of medical education in China, there exists a classification rule that divides the relationship between courses and educational objectives into three categories: “high, medium, and low.” This classification system is intended to ensure that each course directly corresponds to all the educational goals, thereby fostering a more aligned and efficient curriculum. However, it brings forth a series of intricate challenges and dilemmas in practice. One of the primary challenges inherent in this classification rule is the overwhelming workload it imposes on both educators and students. The expectation that every course must address all content related to educational objectives has led to an unrealistic volume of information to cover. This not only hinders the depth of understanding and knowledge retention but also restricts the exploration of critical topics within each subject. This step mirrors the recognition of a problem in the existing curriculum, stemming from an overburdened approach to course content. Another challenge arises from the lack of focus that can result from attempting to cover an extensive range of educational purposes within each course. Instructors are tasked with addressing numerous objectives, risking the dilution of emphasis on core concepts, clinical skills, and critical thinking. The curriculum can become stretched thin, potentially affecting the quality of education provided. This step corresponds to the recognition of the specific needs of learners within the existing system, emphasizing the importance of a more focused approach to course content. The centerpiece of this curriculum overhaul is the CSA classification system—a novel framework that represents far more than a mere categorization of courses. Instead, it serves as a beacon for innovation and adaptation, a responsive approach to the ever-changing educational landscape. This monumental shift is a strategic response to the pressing challenges in medical education, where static, content-focused curricula are giving way to flexible, outcome-oriented paradigms. OBE is the driving force behind this transformation, emphasizing the clear definition of learning outcomes and competencies as the focal point of education. The shift to OBE is nothing short of a pedagogical revolution, designed to empower students by specifying what they should know and be able to do by the end of their educational journey, as shown in Tables 1, 2 for example.

**Table 1:** The roles of Medical Communication Course in achieving Graduate outcomes of basic medical education in the present CSA classification system.

Graduate outcomes of basic medical education		CSA classification
Domains	Items	
1. Science and Scholarship: the medical graduate as a scientist and a scholar	1.1 Possess the fundamental knowledge of the disciplines such as natural sciences, humanities and social sciences and medicine, and apply scientific methods, which will be applicable in future study and medical practices.	A
	1.2 Apply medical and scientific knowledge to individual patients, populations and the health systems.	S
	1.3 Describe the etiology, pathology, natural history, clinical features, diagnosis, treatment and prognosis of common presentations at all stages of life.	
	1.4 Access, critically appraise, interpret and apply evidence from the medical and scientific literature.	
	1.5 Master the basic features of traditional Chinese medicine and its basic principle of diagnosis and treatment.	
	1.6 Apply knowledge of common scientific methods to formulate relevant research questions.	
2. Clinical Practice: the medical graduate as a practitioner	2.1 Conduct effective communications with patients, their family members, colleagues and health professionals of other disciplines.	C
	2.2 Take a medical history in a proper, comprehensive and systematic way.	C
	2.3 Perform a full and accurate physical examination, including a mental state examination, and write medical records as required.	
	2.4 Integrate and interpret findings from the medical history and examination, to arrive at an initial assessment including a relevant differential diagnosis. Discriminate between possible differential diagnoses and propose rational management principles.	
	2.5 Select and justify common investigations, with regard to the pathological basis of disease, utility, safety and cost effectiveness, and interpret the results.	
	2.6 Select and perform a range of common procedures safely.	
	2.7 Make clinical judgements and decisions based on available evidence. Identify and justify relevant management options under the guidance of supervising physicians.	
	2.8 Understand patients’ questions, views, concerns and preferences, and ensure patients’ full understanding of their situations and options. Involve patients in the decision-making and planning of their treatments, including communicating risks and benefits of management options.	C
	2.9 Provide information to patients, and family carers where relevant, to enable them to make fully informed choices among various diagnostic, therapeutic and management options.	C
	2.10 Integrate prevention, early detection, health maintenance and chronic disease management where relevant into clinical practices.	
	2.11 Prescribe medications safely, effectively and economically based on objective evidence.	
	2.12 Recognize and assess deteriorating and critically unwell patients who require immediate care. Perform common emergency and life support procedures.	
	2.13 Describe the principles of end-of-life care for patients, avoiding unnecessary investigations or treatment, and ensuring physical comforts by providing pain relief, psychosocial support and other elements of palliative care.	S
	2.14 Retrieve, interpret and record information effectively in clinical data systems.	
3. Health and Society: the medical graduate as a health advocate	3.1 Accept responsibility to protect and advance the health and well-being of individuals, communities and populations.	
	3.2 Explain factors that contribute to health, illness, disease and success of treatment of populations, including issues relating to health inequities and inequalities, diversity of cultural, spiritual and community values, and socio-economic and physical environment factors.	
	3.3 Communicate effectively in wider roles including health advocacy.	C
	3.4 Explain and evaluate common population health screening and prevention approaches, including the use of technology for surveillance and monitoring of the health status of populations, and provide instructions on patients’ follow-up visits, medications and rehabilitative therapies, etc.	
	3.5 Understand the quality assurance system and safety management system of health care in hospitals, and be aware of their own competence, responsibility and limits in medical practice. Attach importance to patients’ safety, and recognize relevant risk factors in time.	
	3.6 Understand the structures and functions of the national health care system in China, and the roles and relationships between health agencies and services, and understand the principles of rational allocation of resources, to meet the needs of individuals, populations and national health systems.	
	3.7 Understand the global health issues and the determinants of health and diseases.	
4. Professionalism: the medical graduate as a professional	4.1 Provide humanistic and quality health care services to all patients in accordance with the Ethic Principles of Chinese Physicians.	S
	4.2 Demonstrate professional values in health practice, including empathy, respect for all patients and commitment to high quality clinical service standards, and personal qualities of honest, integrity, teamwork, and leadership.	A
	4.3 Explain and apply the main principles of medical ethics in clinical practices. Communicate effectively with patients and their family members, colleagues and other health care professionals regarding ethical issues in medicine	S
	4.4 Be aware of the factors affecting physicians’ health and wellbeing, such as fatigue, stress management and infection control, to mitigate health risks of professional practice, and identify the potential risks posed to patients by their own health.	
	4.5 Abide by the laws and regulations regarding clinical practices well as professional ethics.	
	4.6 Recognize the limits of their own expertise, and show respect for other health care professionals, to learn and work effectively as a team.	
	4.7 Demonstrate awareness of self-directed learning and lifelong learning. Recognize the importance of continuous self-improvement and demonstrate a commitment to excellence.	

**Table 2:** The roles of Medical Communication Course in achieving graduate requirements of basic medical education in the traditional classification system.

Requirement for graduation	The Medical Communication Curriculum
Ethic	M
Laws and regulations	L
Cooperation	M
Basic Sciences	H
Medical foundation	H
Disease diagnosis and treatment	H
Communicative competence	H
Diagnosis and treatment ability	H
Operational capacity	M
Evidence-based ability	H
Preventive care ability	H
Clinic ideation	H
Health care	H
Health system operation	L

The development of the CSA classification system is an integral part of this pedagogical transformation. It acts as a bridge that harmonizes with the OBE framework, creating a dynamic and student-centric curriculum structure. Through CSA, Taizhou University medical education system reimagines how content is delivered and learning objectives are achieved, placing student empowerment and adaptability at the forefront.

By embracing CSA and its principles, Taizhou University is poised to excel in producing healthcare professionals equipped to navigate the complexities of modern healthcare while maintaining a strong foundation in core medical disciplines.

## Data Availability

The raw data supporting the conclusions of this article will be made available by the authors, without undue reservation.
